# Grazing lowers soil multifunctionality but boosts soil microbial network complexity and stability in a subtropical grassland of China

**DOI:** 10.3389/fmicb.2022.1027097

**Published:** 2023-01-05

**Authors:** Leilei Ding, Lili Tian, Jingyi Li, Yujun Zhang, Mengya Wang, Puchang Wang

**Affiliations:** ^1^Guizhou Institute of Prataculture, Guizhou Academy of Agricultural Sciences, Guiyang, Guizhou, China; ^2^College of Life Science, Guizhou University, Guiyang, Guizhou, China; ^3^College of Animal Science, Guizhou University, Guiyang, Guizhou, China; ^4^School of Life Sciences, Guizhou Normal University, Guiyang, Guizhou, China

**Keywords:** fungi, bacteria, grassland, diversity, network complexity, network robustness, niche breadth

## Abstract

**Introduction:**

Long-term grazing profoundly affects grassland ecosystems, whereas how the soil microbiome and multiple soil ecosystem functions alter in response to two-decades of grazing, especially how soil microbiome (diversity, composition, network complexity, and stability) forms soil multifunctionality is rarely addressed.

**Methods:**

We used a long-term buffalo grazing grassland to measure the responses of soil physicochemical attributes, stoichiometry, enzyme activities, soil microbial niche width, structure, functions, and networks to grazing in a subtropical grassland of Guizhou Plateau, China.

**Results:**

The evidence from this work suggested that grazing elevated the soil hardness, available calcium content, and available magnesium content by 6.5, 1.9, and 1.9 times (*p* = 0.00015–0.0160) and acid phosphatase activity, bulk density, pH by 59, 8, and 0.5 unit (*p* = 0.0014–0.0370), but decreased the soil water content, available phosphorus content, and multifunctionality by 47, 73, and 9–21% (*p* = 0.0250–0.0460), respectively. Grazing intensified the soil microbial carbon limitation (+78%, *p* = 0.0260) as indicated by the increased investment in the soil β-glucosidase activity (+90%, *p* = 0.0120). Grazing enhanced the complexity and stability of the bacterial and fungal networks but reduced the bacterial Simpson diversity (*p* < 0.05). The bacterial diversity, network complexity, and stability had positive effects, while bacterial and fungal compositions had negative effects on multifunctionality.

**Discussions:**

This work is an original attempt to show that grazing lowered multifunctionality *via* the reduced bacterial diversity and shifted soil bacterial and fungal compositions rather than the enhanced bacterial and fungal network complexities and stability by grazing. Protecting the bacterial diversity from decreasing, optimizing the composition of bacteria and fungi, and enhancing the complexity and stability of bacterial network may be conducive to improving the soil multifunction of grazing grassland, on a subtropical grassland.

## Introduction

Grassland covers 54% of terrestrial land (Barber-Cross et al., 2022) and occupies ca. 70% of the agricultural land (Abdalla et al., 2018; Ding et al., 2021). It forms ecologically and economically significant functions, including storing one-third of the terrestrial carbon storage (Shrestha et al., 2020; Bai and Cotrufo, 2022), yielding about one-third of terrestrial annual net primary productivity (Ding et al., 2020), supporting livelihoods of 1–2 billion population (Garmendia et al., 2022), and reducing food insecurity (Shrestha et al., 2020). As the most crucial and pervasive anthropogenic disturbance (Wang B. et al., 2019) of grasslands worldwide (Zhang et al., 2018a), and grazing is the major land use accounting for over 22% of the planetary’s land cover (Waters et al., 2019), it is essential to reveal the impact of grazing on grassland for improving grassland management and ecological protection.

A large body of field experiments and meta-analyses have been carried out to measure the influences of grazing on the soil’s physical environment, available elements, and element limitations (Zhang et al., 2018a; Chai et al., 2019; Fenetahun et al., 2021; Liu et al., 2022; Van Syoc et al., 2022; Wang L. et al., 2022). Grazing could decrease soil porosity (Tang et al., 2019) and increase soil bulk density (Binkley et al., 2003; Davidson et al., 2017; Zhang et al., 2018b; Sun et al., 2019; Lai and Kumar, 2020; Fenetahun et al., 2021). These could result in a decline in the air and water permeability (Daniel et al., 2002; Zhang et al., 2018b) and soil moisture (Hou et al., 2014; Li et al., 2021; Liu et al., 2021). However, grazing also increased soil pH (Binkley et al., 2003; Hao and He, 2019; Fenetahun et al., 2021; Li et al., 2021). Since soil pH, O_2_, and water were most crucial driving forces of soil microorganisms (Fierer, 2017), and grazing compressed the living niche for soil microbiome (Chai et al., 2019; Proesmans et al., 2022) and negatively affected the growth of microbes (Zhan et al., 2020), we speculated that the changes caused by grazing may have a far-reaching impact on the attributes of soil microorganisms.

Grazing may not only change the physical environment but also affect nutrient availability. Some studies found that grazing promoted nutrient cycling (Zhang et al., 2021a) and enhanced soil net nitrogen (N) mineralization and N nitrification (Zhou et al., 2017; Dong et al., 2020b), NH_4_^+^ (Lai and Kumar, 2020), NO_3_^–^ (Wang et al., 2016) and available phosphorus (P) (Hao and He, 2019), potassium (K), and calcium (Ca) (Arevalo et al., 1998) and have a positive impact on the availability of soil nutrients (Faghihinia et al., 2020; Xu et al., 2022). Other studies showed that grazing diminished the net mineralization rate (Shan et al., 2011) and decreased available soil N and P (Hao and He, 2019; Liu et al., 2021; Yu et al., 2021), K (Fenetahun et al., 2021), Ca, and magnesium (Binkley et al., 2003). Because nutrient availability has a very important effect in soil microbiome (Fierer, 2017; Wang Z. et al., 2019; Ding et al., 2020), we assumed that the grazing-triggered alterations in available elements may also shift the microbial NP limitations and the attributes of soil microbiome.

Grazing not only changes the soil’s physical environment and nutrient availability but also reduces plant input through reduced aboveground biomass, belowground biomass (Yan et al., 2013; Hao and He, 2019; Wang et al., 2021), plant richness (He et al., 2022), cover, and standing dead and litter (Davidson et al., 2017; Li et al., 2017; Zhao et al., 2021; Rong et al., 2022). Since grassland plants translocated about 30–50% of assimilates below-ground (Yakov and Grzegorz, 2000), grazing could result in lower C availability, which likely aggravates soil microbial carbon limitation and decreases microbial biomass C (Zhou et al., 2017). Since the soil microbial community is fundamentally controlled by C limitation (Ding et al., 2020; Ding and Wang, 2021), we hypothesized that grazing-induced carbon limitation may alter the attributes of soil microorganisms.

Although increasing numbers of studies have reported the influences of grazing on soil’s physical environment, available elements (Fenetahun et al., 2021; Wang L. et al., 2022), and element limitations (Liu et al., 2022), studies about the effects of grazing on the microbial community compositions and the microbial networks are relatively scant (Magalí et al., 2019). Although evidence is mounting that dissecting the relative importance of plants and soils for the impact of the microbiome (Ding et al., 2020; Chen et al., 2021), the linkages of soil physical environment, available elements, and element limitations with the niche width, structure, function, and network of soil microorganism remain relatively scarce, which hinders our understanding of the mechanisms by which grazing changes the soil microorganisms. Soil microorganisms, especially the most frequently studied bacteria and fungi, have an engine effect on multiple ecosystem functions individually (e.g., nutrient transformation) and simultaneously (i.e., multifunctionality, MF) (Ding and Wang, 2021). Grazing strongly alters the biotic diversities and ecosystem functions of grasslands (Zhang et al., 2021b). Previous research studies have typically centered on the influences of diversity and composition on ecosystem functions (Chen et al., 2022; Du et al., 2022). For instance, animal trampling has a major effect on the soil-denitrifying community, which in turn promotes higher denitrification with a higher N_2_O emission (Treweek et al., 2016). Recently, increasing numbers of evidence that highlights ecosystem function are performed *via* the complex network (Chen et al., 2022). Nevertheless, existing studies did not evaluate the relative roles of diversity, composition, and network of soil community on multifunctionality.

Global grasslands are subjected to drastic declines in biodiversity and ecosystem functions (Bai and Cotrufo, 2022), and grazing is the predominant driving factor of the soil microbial community composition (Chen et al., 2021) and function (Eldridge et al., 2016; Xun et al., 2018; Zhang et al., 2021b). Notwithstanding, the underlying driving mechanisms are unclear.

In mountainous regions, since tillage is limited by steep slopes (Hiltbrunner et al., 2012), livestock grazing has the largest deleterious effects on grassland soils, especially in subtropical mountain areas of Guizhou, China. However, evidence supporting the above assumptions remains speculative. The purpose of this study is thus to test the following assumptions: (1) grazing reduces the multiple ecosystem functions, (2) grazing changes the niche width, structure, function, and network of soil microorganisms, (3) grazing-induced alterations in soil physical environment, available elements, and element limitations change the attributes of soil microbiome, and (4) herbivores grazing influences the soil multifunctionality by microbial diversity. This study is the first attempt to systematically tease apart the effects of diversity, composition, and network of soil community on multifunctionality; therefore, it provides a useful basis for understanding grassland grazing.

## Materials and methods

### Sampling sites, design, and soil sampling

The sampling region was in Longli county (N26°19′′44′−26°23′′59′, E106°51′′9′−106°54′′37′, 1490–1620 a.s.l.) of Guizhou Plateau, SW China. This region undergoes a subtropical monsoon humid climate, with an annual average temperature of 14.8°C, the coldest monthly average temperature of 4.6°C, and the hottest monthly average temperature of 23.6°C; The precipitation is abundant, with an annual precipitation of about 1,100 mm, mostly concentrated in summer; the heat is sufficient, the annual sunshine hours are about 1,160 h, and the non-frost period is 283 days. The regional soil type is Haplic alisols (Ding and Wang, 2021), and the dominant species are *Eulalia pallens*, *Arundinella hirta*, and *Carex cruciata wahlenb* (Ding et al., 2020). The natural grassland covers 6,000 hectares (Ding et al., 2020), including grazing lands. The grazing land (50 hectares in this study) is a natural grassland before grazing. The aboveground biomass of grazing land is about three times that of non-grazing land (Xu et al., 2015). The grazing livestock is Qianzhong buffalo. The grazing intensity of the 20-year grazing grassland is one buffalo/hm^2^.

Sampling was carried out at three sampling sites under grazing or ungrazing, respectively, with nearly identical terrain, climate, and soil type (Ding and Wang, 2021), between late September and early October 2017. In total, three 0- to 5-cm soil samples were drilled with stainless steel ring cutting in each site and then mixed into mixed soil samples as a replicate (Ding and Wang, 2021). The mixed soil sample is divided into self-sealed sterile bags, one for the assay of soil moisture, pH, and chemical property, and the other for the assay of soil microorganisms. Soil core was collected from each site using the ring cutting for measuring soil bulk density. Soil hardness was determined using a portable penetrometer (Zhang et al., 2019) (TYD-1, Zhejiang topu Instrument Co., China).

### Assay of soil physicochemical attribute and extracellular enzyme activity

Methods for determining soil physicochemical attributes and extracellular enzyme activity have been described in detail in our recent studies (Ding et al., 2020; Ding and Wang, 2021). Briefly, pH was assayed with 1:2.5 suspension (w/v) (Wang P. et al., 2022). Water content and bulk density were measured oven-dried at 105°C. Organic carbon content was measured using an Elementar analyzer (Bao, 2000). Inorganic carbon content was measured by the hydrogen chloride method (Bao, 2000). Total carbon content = Organic carbon content + Inorganic carbon content. Total nitrogen content was measured by an automatic nitrogen determiner (Bao, 2000). Total phosphorus content was measured by ammonium-molybdate colorimetry (Bao, 2000). Nitrogen availability was measured by the alkali hydrolysis method (Bao, 2000). Phosphorus availability was measured by an ultraviolet spectrometer (Bao, 2000). Potassium availability was measured by a flare photometer (Bao, 2000). Calcium availability and magnesium availability were measured by a spectrophotometer (Bao, 2000). The soil stoichiometric ratios (OC:TN, OC:TP, and TN:TP) were represented as mass ratios. The activities of β-1-4-glucosidase, leucine aminopeptidase, N-acetylglucosaminidase, and acid phosphatase were measured using ELISA test kits of Enzyme-linked Biotechnology Co. (Shanghai, China) (Ding et al., 2020). Vector length and angle were used to quantify the soil microbial carbon limitation and nitrogen/phosphorus limitation based on the four extracellular enzyme activities (Moorhead et al., 2013) using a user-defined function with the setting “trans = 1” (Ding and Wang, 2021).^1^

### DNA extraction, PCR amplification, sequencing, and bioinformatics analysis

This part is performed by Shanghai Major Biotech. Co. (China) (Ding et al., 2020). Briefly, soil DNA was extracted using a DNA isolation kit, and its quality and quantity were checked using 0.8% agarose gel electrophoresis. The 16S rRNA gene V4 region was amplified using the primers 515F and 907R, and the fungal rRNA gene ITS region was amplified with the primers ITS3_KYO2 and ITS4 (Ding et al., 2020). The Hiseq 2500 platform (PE250 mode) was used for sequencing. After the filtering, splicing and quality control of raw reads were performed using FLASH (Magoc and Salzberg, 2011) and UCHIME (Edgar et al., 2011) software, and the effective tags were clustered into an OTU using USEARCH (Edgar, 2010) with a cut-off of 97%. The OTU was named and the diversity indices (observed species, Shannon, Simpson, Chao1, and goods coverage) were produced by USEARCH (Edgar, 2010). The function of the soil bacterial community was annotated using FAPROTAX v1.2.6 (Functional Annotation of Prokaryotic Taxa) (Louca et al., 2016) and the “tax4fun” package (Asshauer et al., 2015). The function of the soil fungal community uses the “FUNGuildR” package^2^ in Rv3.6.1.^3^

### Quantification of soil multifunctionality

Soil multifunctionality is defined as the synthesis of multiple supporting ecosystem properties at a small scale (Ding and Wang, 2021), and it can provide an insight into the overall change of multiple soil properties. A total of three complementary methods [the entropy-based multifunctionality when considering a maximum number of functions and considering the increasing number of functions (Ding and Wang, 2021) and the threshold approach-based multifunctionality (Byrnes et al., 2014; Ding and Wang, 2021)] were used to quantify the soil multifunctionality based on the following soil properties: AN, AP, AK, ACa, AMg, pH, WC, BD, SH, βGC, NAG, LAP, ACP, C limitation, and vector angle (NP limitation). These variables were included in soil multifunctionality since they either measure real soil functions or are good proxies of soil functioning (Delgado-Baquerizo et al., 2017; Bagousse-Pingueta et al., 2019; Chen et al., 2020a,b). Inherent soil properties often define the soil’s basic functions (Amsili et al., 2021). Soil pH can be considered as the result of the complex interaction of various soil materials, such as nitrogen, phosphorus, potassium, calcium, magnesium, and organic materials (Xu et al., 2006; Jiang et al., 2015; Neina, 2019; Penn and Camberato, 2019). The changes in soil BD can reflect the changes in organic matter content, porosity, and compaction (Aşkin and Özdemir, 2003; Pravin et al., 2013), especially under long-term grazing. SH is a soil mechanical property comprehensive reflection of soil organic matter, humus, water content, texture, and structure. SH was determined using a portable penetrometer (Zhang et al., 2019) and reflected the degree of difficulty of root and water penetration (Ono et al., 2020). In addition, other soil properties are often included in soil multifunctionality (Byrnes et al., 2014; Ding and Wang, 2021; Du et al., 2022; Wang P. et al., 2022). Before calculating soil multifunctionality, the C limitation, SH, and BD were reflected using r(f) = –f + max(f), and the vector angle was reflected using f(x) = x−45 when the value of the vector angle was <45 (indicating N limitation) and was reflected using f(x) = −x + 45 when the value of vector angle was >45 (indicating P limitation). By doing so, the high value corresponds to the desired good value (Byrnes et al., 2014; Ding and Wang, 2021).

### Statistical analysis

Kruskal–Wallis test, Wilcox test (William, 1971; Myles and Douglas, 1973), or *t*-test (Zar, 2014) was applied to test the significance of the difference in soil physicochemical attributes, stoichiometric ratios, enzyme activities, C/NP limitations, and multifunctionalities. Principal component analysis (PCA) and non-metric multidimensional scaling (NMDS) (Oksanen et al., 2019) analysis were applied to distinguish the soil microbial community between grazing and ungrazing. Linear discriminant analysis (LDA) effect size (LEfSe) analysis (Segata et al., 2011) was applied to identify the differential taxa between grazing and ungrazing. Microbial network analysis was used to reveal the microbial interaction strength at the threshold of Pearson correlations *r* > 0.8 and the Benjamini and Hochberg corrected *p* < 0.01 using the “igraph” package (Csardi and Nepusz, 2006) in R v4.05.^4^ Kolmogorov–Smirnov test (William, 1971) was used to compare the distribution of node degree, closeness, transitivity, and eigenvector centrality of the microbial network between grazing and ungrazing. Kolmogorov–Smirnov test and Kruskal test (William, 1971) were run to compare the distribution and difference in the microbial network stability (average degree and network connectivity) after 50% nodes were randomly removed. Microbial network stability was visualized using network robustness analysis that shows the declines in microbial average degree and natural connectivity with the increasing proportion of removing nodes or edges (Fan et al., 2018). The subnetwork attributes are extracted for each sample using the “igraph” package (Csardi and Nepusz, 2006). Niche breadth was calculated using the “spaa” package (Zhang, 2016) and was tested using the Wilcox test (Myles and Douglas, 1973), and the dispersal–niche continuum index was applied to quantify the relative role of niche or dispersal process for understanding the drivers that shape community assembly (Vilmi et al., 2020). Zero- and first-order Pearson correlation analyses were used to test the linkages of the available element, physical environment, element limitation with niche width, soil microbial diversity, composition, network complexity and stability, the linkages of soil microbial diversity, composition, and network complexity and stability with multifunctionality using the “ppcor” package (Kim, 2015) in R3.6.1. Before Pearson correlation analysis, the first principal component (PC1) of PCA was used to reduce multiple variables into a single virtual variable (available element, physical environment, bacterial network complexity, and fungal network complexity), which covers more than 96.7% of the original information. The composition was indicated by NMDS1. The “ggplot2” package in R and Gephi 0.9.7^5^ were applied to visualize the results.

## Results

### Soil properties and multifunctionality under grazed and ungrazed

**FIGURE 1:**
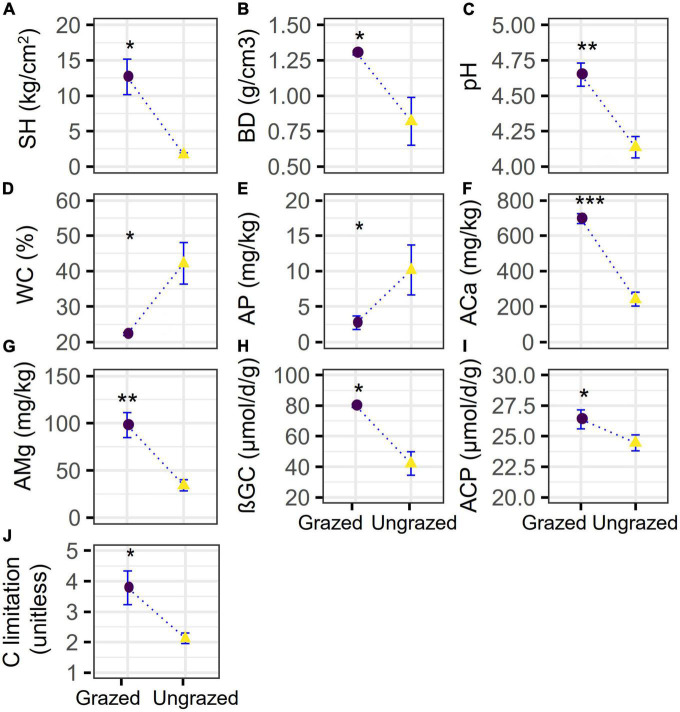
Soil physical, chemical property, enzyme activity, and carbon limitation under grazed and ungrazed. The differences in **(A)** soil hardness (SH), **(B)** soil bulk density (BD), **(C)** pH, **(D)** water content (WC), **(E)** phosphorus availability (AP) content, **(F)** calcium availability (ACa) content, **(G)** magnesium availability (AMg) content, **(H)** β-1-4-glucosidase (βGC), **(I)** acid phosphatase (ACP), and **(J)** microbial carbon limitation between grazed and ungrazed. *p < 0.05, **p < 0.01, and ***p < 0.001.

**FIGURE 2:**
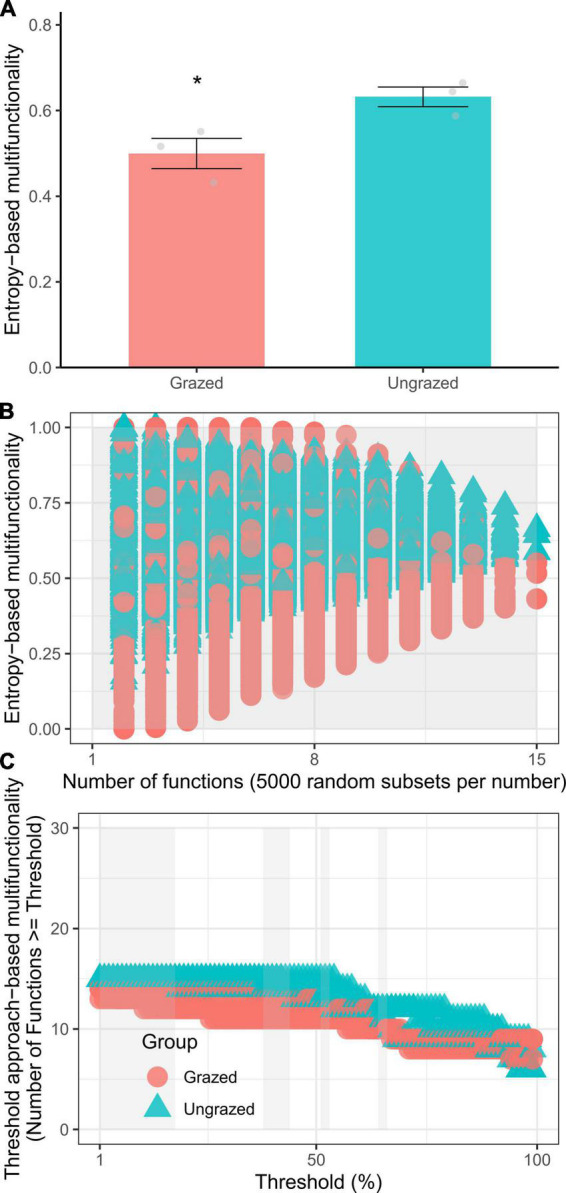
The entropy-based multifunctionality [**(A)**, considering maximum number of functions; **(B)**, considering the increasing number of functions] and threshold approach-based multifunctionality **(C)** of soils under grazed and ungrazed. *p < 0.05.

Compared with the ungrazing, grazing significantly increased the soil SH, ACa, and AMg by +6.5 (*p* = 0.0160), +1.9 (*p* = 0.00015), +1.9 (*p* = 0.0064) times and elevated soil BD, pH, ßGC, ACP, and microbial C limitation by +59% (*p* = 0.0370), +0.5 unit (*p* = 0.0014), +90% (*p* = 0.0120), +8% (*p* = 0.0310), and +78% (*p* = 0.0260), respectively, whereas ungrazing decreased the soil WC and AP by −47% (*p* = 0.0270) and −73% (*p* = 0.0250), respectively (Figure 1). However, statistical differences were not observed in other soil properties (*p* = 0.10–1) (Supplementary Figure 1). Besides, the grazing significantly increased the entropy-based multifunctionality by −21% when considering the maximum number of functions (*t*-test, *p* = 0.0435) and by −13.8–20.9% when considering the increasing number of functions (*t*-test, *p* = 0 – 1.5e−144). The grazing also significantly increased the threshold approach-based multifunctionality by −9 to −14% (Kruskal–Wallis test, *p* = 0.0340–0.0460, Figure 2), supporting our first hypothesis.

### Soil microbial structure under grazed and ungrazed

**FIGURE 3:**
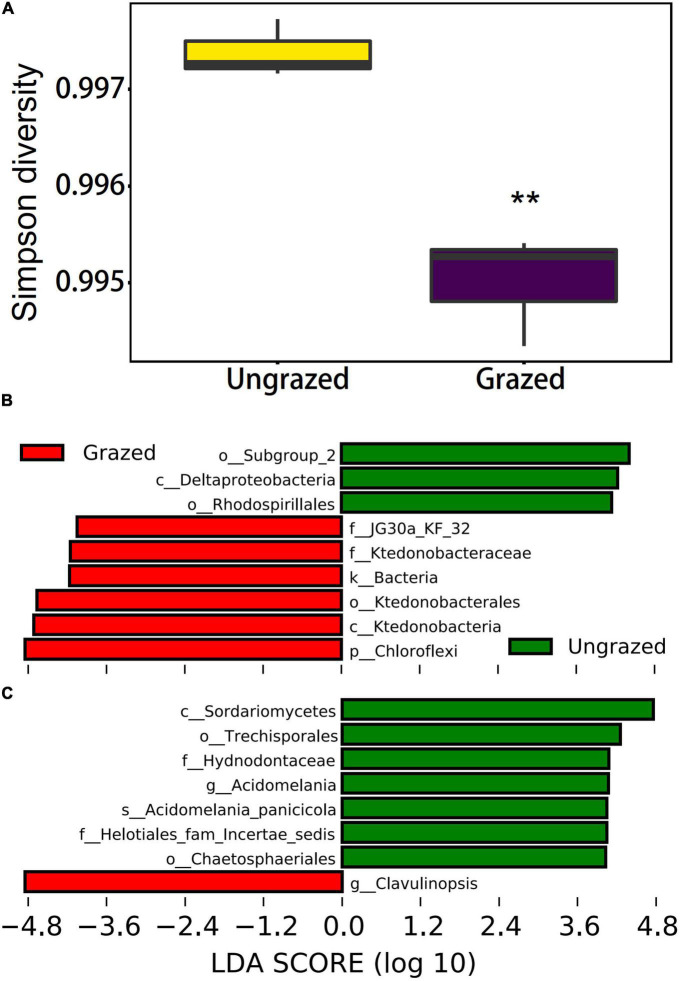
Soil bacterial diversity **(A)** under grazed and ungrazed, and LEfSe analysis for bacterial **(B)** and fungal **(C)** communities showing the significantly enriched taxa under grazed and ungrazed. **p < 0.01.

As expected, grazing significantly reduced the bacterial Simpson diversity by −0.24% (*p* = 0.0079, Figure 3A). Unexpectedly, no statistical differences were found in fungal diversity between grazed and ungrazed (*p* > 0.05, Supplementary Figure 2). However, PCA and NMDS showed that there was a clear distinction between the grazed and the ungrazed (Supplementary Figure 3). Furthermore, LEfSe analysis for bacterial communities showed that *Subgroup_2* order belonging to *Acidobacteria* phyla and *Rhodospirillales* order and *Deltaproteobacteria* class belonging to *Proteobacteria* phyla were evidently enriched in the ungrazed soils; *Ktedonobacteria* class, *Ktedonobacterale*s order, *JG30a_KF_32* family, *Ktedonobacteraceae* family belonging to *Chloroflexi* phyla, and *Chloroflexi* phyla were evidently enriched in the grazed soils (LDA score > 3.6, Figure 3B). LEfSe analysis for fungal communities showed that *Helotiales_fam_Incertae_sedis* family, *Acidomelania* genus, *Acidomelania_panicicola* species, *Sordariomycetes* class, and *Chaetosphaeriales* order, which belong to *Ascomycota* phyla, *Trechisporales* genus, and *Hydnodontacea* family, which belong to the *Basidiomycota* phyla were evidently enriched in the ungrazed soils. The *Clavulinopsis* genus belonging to *Ascomycota* phyla was evidently enriched in the grazed soils (LDA score > 3.6, Figure 3C).

### Soil microbial putative functions under grazed and ungrazed

Compared with the ungrazed, the grazed significantly decreased the relative abundance of intracellular parasites, non-photosynthetic cyanobacteria, anoxygenic photoautotrophy S oxidizing, and methanol oxidation (Welch’s *t*-test with two sides *p* = 1.62e−3 to 0.034, Supplementary Figure 4A). The grazed significantly decreased the relative abundance of C fixation genes (accA, accB, and FBP), lignin decomposition gene (tyrosinase), starch decomposition gene (bglA) chitin decomposition gene (E3.2.1.96), nitrogen fixation gene (nifD), denitrification gene (napB, NO_3_^–^→N_2_), and antibiotic resistance gene subtypes (ampG, emrA, and acrA) and significantly increased the relative abundance of C fixation genes (frdA, frdB, frdC, and gap2), antibiotic resistance gene (ARGs) type (bacitracin), and ARGs subtypes (bceS and lmrP) (Welch’s *t*-test with two sides *p* = 4.59e−3 to 0.049, Supplementary Figure 4B). The fungal functional analysis showed that the grazed significantly decreased the relative abundance of two growth forms (Thallus and Polyporoid, Welch’s *t*-test with two sides *p* = 0.035–0.036) and three guilds (Wood Saprotroph, Lichen Parasite, and Orchid Mycorrhizal, Welch’s *t*-test with two sides *p* = 0.041–0.044, Supplementary Figure 4C), whereas insignificant differences were found in the relative abundance of plant pathogen fungi, arbuscular mycorrhizal fungi, and ectomycorrhizal fungi between the grazed and the ungrazed (Welch’s *t*-test with two sides *p* = 1, 1, 1).

### Soil microbial networks under grazed and ungrazed

**FIGURE 4:**
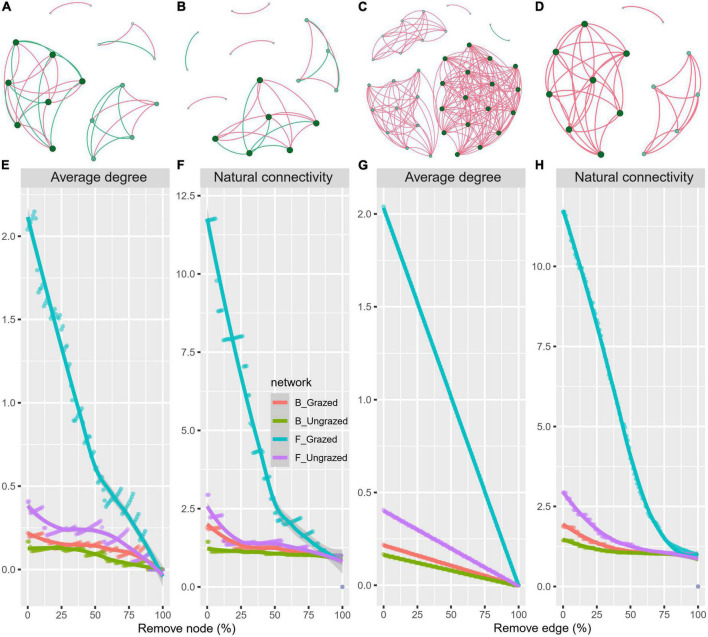
Soil bacterial **(A,B)** and fungal **(C,D)** co-occurrence networks under grazed **(A,C)** and ungrazed **(B,D)**, and network robustness analysis **(E–H)** for microbial communities between the grazed and ungrazed. A node suggests an individual OUT, its color and size are proportional to its degree; a link represents the significant Pearson correlations with *r* > 0.8 and the Benjamini and Hochberg corrected *p* < 0.01. A red line suggests a positive relationship, but a blue line suggests a negative relationship. Network robustness analysis showing smaller decline at the same proportion indicates more stability within networks.

To decipher the influences of grazing on microbiome associations, the bacterial and fungal networks under the grazing and ungrazing treatments were built. Different association patterns were observed (Figures 4A–D), supporting our second hypothesis. Compared to the ungrazed, the grazed increased the number of edges (by +40.00%, +4.23 times), average degree (+48.24%, +1.06 times), natural connectivity (+1.01 and +4.52 times), and the number of negative edges (+10 unit and +1 unit) and negative edge proportion (+0.1829 unit and +0.0049 unit) of bacterial and fungal networks, whereas decreased the positive edges proportion (28.57%, 0.49%) and vulnerability (−39.05%, −48.86%) of bacterial and fungal networks. The grazed decreased the number of nodes (by −5.56%), number of clusters (−33.33%), centralization degree (−10.00%), center eigen (−11.11%), and modularity (−4.51%) of the bacterial network; however, increased those (+153.33, +21.87, 66.67, +10.64, +8.33, and +18.66%) of fungal networks. The grazed increased the connectance (+57.50%) of the bacterial network, whereas decreased that (−21.87%) of the fungal network. The grazed did not change the number of positive edges of the bacterial network (0%), but increased that of the fungal network (+4.21 times) (Supplementary Table 1). Kolmogorov–Smirnov test indicated that the node degree, closeness, transitivity, and eigenvector centrality under the grazed were statistically distinct from those under ungrazed (*p* = 0–0.0001, Table 1). We assessed the difference in network stability (average degree and network connectivity) between the grazed and ungrazed treatments by network bootstrapping after 50% of nodes were randomly removed. Kolmogorov–Smirnov test indicated that the grazed significantly changed the network stability (average degree, *p* = 0 and network connectivity, *p* = 0), and the Kruskal test revealed that the average degree and network connectivity of the fungal network were 1.36–9.30-folds those of bacterial network, regardless of the grazed and ungrazed (Table 2). Interestingly, the grazed soils significantly increased the average degree and network connectivity of the bacterial network by +30.37% (*p* = 0) and +4.01 times (*p* = 0) and those of the fungal network by +9.46% (*p* = 0) and + 2.65 times (*p* = 0), respectively. Furthermore, we performed the robustness analysis of networks based on removing an increasing proportion of nodes and edges. The results showed that the average degree and network connectivity of the fungal network were higher than those of the bacterial network, and those of the grazed were higher than those of the ungrazed, irrespective of the remove of nodes and edges (Figures 4E, F).

**TABLE 1:** Results of the Kolmogorov–Smirnov test comparing bootstrapped node attributes of networks under grazed and ungrazed.

Comparison	Degree	Closeness	Transitivity	Eigenvector centrality
Grazed B vs. Ungrazed B	0.4133****	0.4444****	0.3281****	0.2443****
Grazed F vs. Ungrazed F	0.7109****	1****	0.0283****	0.4183****
Ungrazed B vs. Ungrazed F	0.5348****	0.8689****	0.3097****	0.3089****
Grazed B vs. Grazed F	0.7109****	1****	0.0099***	0.2611****

For each network, node attributes were computed by bootstrapping 100,000 times. Kolmogorov–Smirnov test compares the cumulative distribution of two properties where the null hypothesis is that the properties have same distribution patterns. The values in each cell represents the maximum difference in the absolute cumulative distribution function. ***P < 0.001, ****P < 0.0001. B, bacteria; F, fungi.

**TABLE 2:** Results of the Kolmogorov–Smirnov test and Kruskal–Wallis test comparing network stability (average degree and network connectivity) of networks under grazed and ungrazed after 50% nodes were randomly removed.

Method	Comparison	Average degree	Natural connectivity
Kolmogorov–Smirnov test	Grazed B vs. Ungrazed B	0.2442****	0.2835****
	Grazed F vs. Ungrazed F	0.9822****	0.9579****
	Ungrazed B vs. Ungrazed F	0.6554****	0.6074****
	Grazed B vs. Grazed F	0.9994****	0.9909****
Kruskal–Wallis test	Grazed B vs. Ungrazed B	0.1322 ± 0.0004 vs. 0.1014 ± 0.0003****	1.2514 ± 0.0014 vs. 1.1433 ± 0.0008****
	Grazed F vs. Ungrazed F	1.2298 ± 0.0021 vs. 0.2456 ± 0.0007****	5.6659 ± 0.0107 vs. 1.5511 ± 0.0029****
	Ungrazed B vs. Ungrazed F	0.1014 ± 0.0003 vs. 0.2456 ± 0.0007****	1.1433 ± 0.0008 vs. 1.5511 ± 0.0029****
	Grazed B vs. Grazed F	0.1322 ± 0.0004 vs. 1.2298 ± 0.0021****	1.2514 ± 0.0014 vs. 5.6659 ± 0.0107****

For each network, node properties were computed by bootstrapping 100,000 times. The values in top four cells represent difference for Kolmogorov–Smirnov test, which the maximum difference in the absolute cumulative distribution function; The values in bottom four cells represent mean value ± 95% confidence interval for Kruskal–Wallis test. ****P < 0.0001. B, bacteria; F, fungi.

### Niche breadth and assembly mechanism of microbial communities

Wilcox test revealed that compared with the ungrazed, the grazed decreased the niche breadth index of bacteria and fungi by −10.43% (*p* < 2.22e−16) and −3.88% (*p* < 2.22e−16), respectively (Supplementary Figure 5A). This supported our second hypothesis. The dispersal–niche continuum index showed that the *E*-values from the niche-controlled model were clearly lower than those from the niche– and dispersed-controlled model and dispersal-controlled model (Supplementary Figures 5B, C), indicating that niche and dispersal limitations were the main driving force of bacterial and fungal community assembly.

### The relationships of driving forces and soil microbial structure and network

The zero-order Pearson correlation analysis showed that fungal composition (indicated by NMDS1) was not significantly related to C limitation (Supplementary Figures 6A, B), and the vulnerability of the fungal network was significantly related to AP, pH, and BD (Supplementary Figure 6B). Other zero-order relationships were significant (Supplementary Figures 6A, B). The first-order Pearson correlation analysis demonstrated that soil microbial composition and network were evidently associated with the available element, physical environment, C limitation, and niche width (Supplementary Figures 6C–I).

## Discussion

### Grazing changed the soil’s physical property, chemical property, and ecto-enzyme activity

This study found that grazing evidently elevated the SH (Shan et al., 2011; Zhang et al., 2019; Li et al., 2021; Liu et al., 2021), BD, pH (Blank et al., 2006; Ajorlo et al., 2011; Rong et al., 2022), ACa, AMg (Jusoff, 1988; Blank et al., 2006), ßGC, ACP, and C limitation (Goenster-Jordan et al., 2021), but decreased the WC and AP (Niu et al., 2016; Sigcha et al., 2018; Chen et al., 2021; Fenetahun et al., 2021; Rong et al., 2022; Figure 1). The buffalo squeezed the soil pores that include those pores originally storing water and air, through treading, which caused soil compaction (Ludvíková et al., 2014), elevated the bulk density (Li et al., 2008; Zhan et al., 2020; Fenetahun et al., 2021; Wade et al., 2022) and soil hardness, and reduced soil water infiltration (Daniel et al., 2002). Soil compaction is a global environmental issue of increasing importance occurring in grasslands and other lands (Anneke et al., 2010). Furthermore, buffalo decreased the vegetation cover (Wang and Tang, 2019) through eating and treading and enhanced the evaporation of soil water to air (Zhan et al., 2020). All finally depleted the soil water content (Hao and He, 2019; Tang et al., 2020). The returns of ACa and AMg in form of dung and urine (Saunders, 2012) and accelerated soil weathering by grazing (Lin et al., 2021) could elevate the availability of calcium and magnesium, which, in turn, are conducive to improving soil pH (Figure 1C). Decreases in plant biomass and coverage (Wang and Tang, 2019; Lin et al., 2021) and removal of aboveground biomass by buffalo eating and treading, and root biomass (Wade et al., 2022) due to the depletion of soil water content, and increased bulk density (Lin et al., 2021) and soil hardness (Figure 1A), generally reduced the C inputs, which, in turn, commonly reduced the bioavailable soil C (Ravhuhali and Moyo, 2021), aggravated C limitation (Goenster-Jordan et al., 2021) as indicated by the increased investment in C-capturing enzyme (ßGC, Figure 1H), which can hydrolyze sugars and release labile C. The vector angle under ungrazed was <45, indicating that the soil microbial communities were limited by N; the vector angle under grazed crossed with 45, indicating that the soil microbial communities were under N and P co-limitation. This showed that grazing shifted soil microbial communities from under N limitation to under NP limitation. The decrease of AP explained the P limitation, and this resulted in the increased investment of P-capturing enzyme (ACP, Figure 1I). In brief, grazing changed the soil’s physical and chemical properties and ecto-enzyme activities and exacerbated microbial C limitation.

### Grazing altered soil microbial niche width, structure, and functions

Decreases in soil microbial niche width could be driven by soil available elements and physical environment rather than C limitation, and the interconnections were further evidenced by the first-order Pearson correlation analysis (Supplementary Figures 7A–D). This supported our third hypothesis. The negative correlation between soil available elements and bacterial-/fungal-niche width (i.e., the zero-order correlation with Pearson’s *r* = −0.99) slightly weakened after removing the effect of C limitation (*r* = −0.95) and physical environment (*r* = −0.97). The negative effect of pH was weakened (from *r* = −0.97 to −0.91) if the effect of the C limitation was controlled. So were the effect of BD (from *r* = −0.93 to −0.91) and WC (from *r* = 0.95 to 0.89) if the effect of ACa was controlled. In addition, the negative effect of ACa was maintained or weakened after removing the effect of C limitation, SH, BD, WC, pH, and AMg.

As the consequences of the reduction of niche width (r of zero-order Pearson correlation between niche width and microbial composition = −0.97 to −0.99), bacterial and fungal communities under grazed were distinct from those under the ungrazed (Rong et al., 2022; Wang L. et al., 2022). Interestingly, shifts in bacterial composition could be driven by the soil’s physical environment and available elements (Supplementary Figures 6A, C, 7E). The positive effect of soil physical environment, available elements, and C limitation was weakened (from *r* = 0.97, 0.98, 0.87–0.90, 0.94 ns) after removing the effect of C limitation (Supplementary Figures 6A, C). The positive effect of soil pH and ACa was weakened (from *r* = 0.97, 0.97 to 0.89–0.93) after removing the effect of C limitation, BD, and AP (Supplementary Figures 6B, 7E). Consistent with previous findings, grazing maintained the fungal diversity (Qin et al., 2021) but declined the bacterial diversity (Chen et al., 2021; Foley et al., 2022). This indicated that the bacterial community and fungal community have distinct responses to grazing in terms of diversity. Interestingly, recent work suggested that light grazing intensity raised the soil microbial α-diversity, while high grazing intensity decreased diversity, whereas moderate grazing maintained a soil microbial diversity level close to that of ungrazing (Xun et al., 2018). This may suggest that in this study, grazing is the moderate intensity for fungi, while it is high intensity for bacteria. Intriguingly, the negative effect of soil available elements on the bacterial Simpson diversity was weakened (from *r* = −0.95 to −0.90) after removing the effect of C limitation (Supplementary Figures 6A, I). The negative effect of SH was weakened (from *r* = −0.98 to −0.89 to −0.96) after removing the effect of C limitation, BD, WC, pH, AMg, and AP. The negative effect of ACa was weakened (from *r* = −0.95 to −0.88 to −0.90) after removing the effect of C limitation and AMg (Supplementary Figures 6B, 7K).

Shifts in fungal composition could be driven by soil C limitation and available elements. The positive effect of the soil’s physical environment was weakened (from *r* = 0.95 to ns), and the positive effect of the soil’s available elements was enhanced (from *r* = 0.95 to 0.97) after removing the effect of C limitation (Supplementary Figures 6A, D). The effect of the C limitation was also enhanced after removing the effect of available elements (from *r* = 0 to −0.89) and ACa (from *r* = 0 to −0.88). The positive effect of soil pH and ACa was slightly weakened and enhanced (from *r* = 0.97, 0.95 to 0.92, 0.97) after removing the effect of the C limitation. The positive effect of soil pH was slightly weakened (from *r* = 0.97 to 0.89–0.91) after removing the effect of AMg and AP (Supplementary Figures 6B, 7F).

The shifts in microbial composition might have important implications for soil element cycling. Interestingly, contrary to our expectation that the genes responsible for regulating C and N cycling were heightened by grazing in Qinghai-Tibetan Plateau (Dong et al., 2020a), the grazing might slow the anoxygenic photoautotrophy S oxidizing and methanol oxidation (Supplementary Figure 4A), probably owing to an oxygen-limiting condition induced by grazing-driven soil compaction (Tang et al., 2020). Grazing in Qinghai-Tibetan Plateau also fasted C fixation (accA and frdA) and degradation (mnp, apu, and amyA), CH4 metabolism (pmoA and mxa), the ammonia-oxidizing (amoA2), nitrification (hao), and denitrification (nirS3 and nirK1) processes (Dong et al., 2020a); however, in Guizhou plateau, grazing might slow C fixation (accA, accB, FBP) and lignin decomposition (tyrosinase), starch decomposition (bglA) and chitin decomposition (E3.2.1.96), nitrogen fixation (nifD), and denitrification (napB) while grazing might fast other C fixation processes (frdA, frdB, frdC, and gap2) (Supplementary Figure 4B). Grazing intensities, climate, soil type, and vegetation are often used to explain this inconsistency. Denitrification is a microbially mediated stepwise reduction process. In this process, NO_3_^–^ was reduced gradually to NO_2_^–^, NO, N_2_O, and finally N_2_. This process can leak N_2_O. Since N_2_O has a global warming potential nearly 296 times higher than that of CO_2_ (Fang et al., 2020), grazing-induced declines of denitrification may be beneficial to the emission reduction of N_2_O. In addition, grazing-induced declines of denitrification may help to unchanged TN and AN (Supplementary Figures 1D, F).

Furthermore, the decreases in the relative abundance of two growth forms (Thallus and Polyporoid) and three guilds (Wood Saprotroph, Lichen Parasite, and Orchid Mycorrhizal funfus) induced by grazing (Supplementary Figures 4F, G) may be attributed to the reduction of litter and shifts in plant hosts (declined in the Lichen and Orchid) by grazing or tramping (Kuske et al., 2012). Besides, some implications for soil health have also been observed. On the one hand, intracellular parasites represent a diverse and widely distributed group of pathogenic microbes related to some acute and chronic diseases,^6^ and the grazing decreased the relative abundance of intracellular parasites, indicating that grazing improved soil health. On the other hand, the propagation of ARGs has been widely perceived as a great threat to global public health. Grazers could propagate ARGs to clinical pathogens (Du et al., 2020) and soils. In the Eurasian steppe, grazing reduced or did not alter ARGs (Du et al., 2020; Zheng et al., 2021). In this work, the grazing decreased the resistance to Beta-Lactam (ampG) and Multidrug (emrA and acrA) subtypes, whereas increased the relative abundance of one ARGs type (bacitracin), Bacitracin (bceS), and Multidrug (lmrP) subtypes (Supplementary Figures 4D, E). Although the transmission mechanism of ARGs in grazing soils remains unknown, the focus should be centered on the grazing system, which may enrich ARGs and threaten the health of soil and human beings dependent on these soils, especially when the influence of grazing on the diversity and abundance of soil ARGs are controversy (Du et al., 2020; Zheng et al., 2021).

### Grazing changes soil bacterial and fungal networks

Beyond shifts in microbial community composition, grazing also altered the potential interaction patterns of soil microbiome (Magalí et al., 2019; Chen et al., 2021), which supported our second hypothesis. The increases in the average degree and network connectivity of fungal networks in response to the grazing were greater than those of bacterial networks (Figure 4), indicating that the fungal network was more sensitive to grazing than the bacterial network (Chen et al., 2021). Contrary to the previous findings that grazing increased positive network links (Magalí et al., 2019), in this work, both the bacterial network’s and fungal network’s positive links were slightly reduced by grazing (Supplementary Table 1). This is contrary to the stress-gradient hypothesis (Ding and Wang, 2021), which suggests that cooperative interactions are increased in a community, and this is a survival strategy for soil microbial communities under grazing stress (Magalí et al., 2019). Nonetheless, grazing might enhance the selection for microbes with identical niche demand, intensifying competition among microbes (Eldridge et al., 2017) and ultimately showing an increase in negative links within the community (Figures 4A–D and Supplementary Table 1), further suggesting that grazing boosted network complexity. Since positively correlated microorganisms are more likely to be suppressed by the same interference than negatively correlated microorganisms, this negative links may also be the basis for grazing to enhance the stability of the network. Our study provides an alternative scenario different from the previous study, and this may enhance an understanding of the influences of grazing on microbial networks. Because the negative correlation has a great effect on ecosystem function (Romdhane et al., 2022), the negative correlation may have unknown enlightenment on the soil function of grazing grassland that needs further study.

Soil microbial network complexity (indicated by PC1 of subnetwork attributes) could be mainly driven by the soil’s physical environment. The negative effect of the physical environment and available element on soil bacterial network complexity was maintained when controlling for the NP limitation (from *r* = −0.97, −0.90), whereas the negative effect of the physical environment was weakened (from *r* = −0.97 to −0.93) when controlling for the C limitation (Supplementary Figures 6A, B, E, F). The negative effect of SH (*r* = −0.88), BD (*r* = −0.94), pH (*r* = −0.97), AMg (*r* = −0.88), and ACa (*r* = −0.90) was maintained, and the positive effect of WC (from *r* = 0.97 to 0.98) and AP (from *r* = 0.93 to 0.98) was enhanced when controlling for the NP limitation; however, the negative effect of pH (from −0.97 to −0.92) and the positive effect of WC (from *r* = 0.97 to 0.92) was weakened when controlling for the C limitation. Unexpectedly, the C limitation and the NP limitation did not significantly impact the bacterial network complexity. Interestingly, although on the whole level, physical environment, available element, C limitation, and the NP limitation did not significantly impact the fungal network complexity (*p* = 1), the effect of AMg was enhanced (from ns to *r* = 0.98) when controlling for the C limitation at the single variable level. More interestingly, the effect of C limitation was enhanced (from ns to *r* = −0.97) when controlling for the AMg. Besides, the effect of pH was also enhanced (from ns to *r* = 0.88, 0.92, 0.91) when controlling for the C limitation, SH, and ACa (Supplementary Figures 6B, 7G, H).

Soil microbial network stability (indicated by network vulnerability) could be mainly driven by the soil’s physical environment. The negative effect of the soil’s physical environment on the bacterial network vulnerability was weakened (from *r* = −0.99 to −0.89 to −0.95) after removing the effect of C limitation and available element (Supplementary Figures 6A, G, H). The negative effect of SH was enhanced and weakened after removing the effect of BD (from *r* = −0.97 to −0.99) and WC (from *r* = −0.97 to −0.88), respectively. The negative effect of BD on the bacterial network vulnerability was weakened (from *r* = −0.97 to −0.88 to −0.91) after removing the effect of ACa (from *r* = −0.97 to −0.91) and AMg (from *r* = −0.97 to −0.88). The negative effect of BD (from *r* = −0.97 to −0.89 to −0.99) and the positive effect of WC (from *r* = 0.97 to 0.90) were weakened after removing the effect of C limitation, SH, and AP. The positive effect of one driving force on the fungal network vulnerability was immensely weakened (from *r* = 0.88–0.92 to ns) after removing the other driving forces. The positive effect of SH (from *r* = 0.91 to 0.94) and ACa (from *r* = 0.95 to 0.91) was enhanced and weakened after removing the effect of pH (Supplementary Figures 6B, 7I, J).

### Multifunctionality is linked to soil microbiome

**FIGURE 5:**
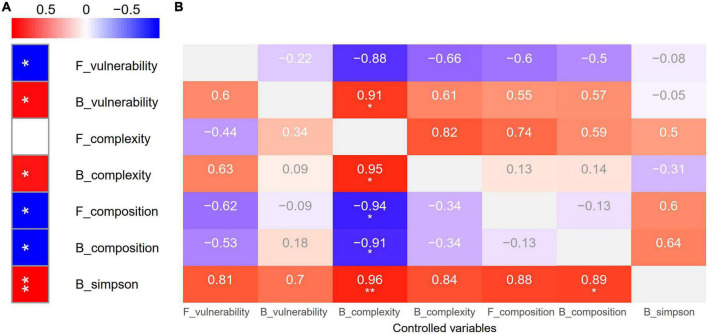
Zero-order **(A)** and first-order **(B)** Pearson correlation analysis showing the relationships of biotic driving forces and soil multifunctionality. *p < 0.05, **p < 0.01.

In line with previous findings, grazing had a negative impact on multifunctionality (Zhang et al., 2015; Eldridge et al., 2016; Zhang et al., 2021b). This adverse effect could be attributed to the response of soil microbiome (Chen et al., 2021). Traditional classical studies have widely confirmed that diversity is the strongest driving force of multifunctionality (van der Plas, 2019). Nevertheless, increasing numbers of studies have found that the complexity of biotic networks dominates multifunctionality (Jiao et al., 2019; Wagg et al., 2019). However, grazing-induced changes in soil microbial community composition drove soil function (Fan et al., 2021a,b). But a recent finding indicated that it is not diversity loss induced by anthropogenic pressures but the shifts in community composition and the declines in microbial abundance altered soil function. Anthropogenic pressures also eliminated biodiversity effects *via* the shifted community composition and the declined abundance (Yang et al., 2022). Our study first dissected the roles of soil microbial diversity, composition, network complexity, and stability on multifunctionality. The positive effect of bacterial network stability was maintained and the negative effect of fungal network stability was tremendously weakened (from *r* = 0.91, −0.88 to 0.91, ns) after removing the effect of fungal network complexity (Figure 5). Interestingly, the positive effect of bacterial network complexity was enhanced (from *r* = 0.86 to 0.95) after removing the effect of fungal network complexity, whereas fungal network complexity has never played a significant role (*r* = −0.44–0.82, *p* = 0.09–1). Importantly, the negative effects of bacterial and fungal compositions were enhanced (from *r* = −0.87, −0.87 to −0.91, −0.94) after removing the effect of fungal network complexity. More importantly, the positive effect of bacterial diversity was maintained and reduced (from *r* = 0.96 to 0.96, 0.89) after removing the effect of fungal network complexity and bacterial composition (Figure 5). This shows that our data support the traditional view and our fourth hypothesis; nevertheless, the network complexity also plays an important role to link the soil microbiome and multifunctionality, which also supports that the simplification of soil complex network links could debilitate ecosystem function (Jiao et al., 2019; Wagg et al., 2019). Interestingly, network complexity was enhanced while multifunctionality was impaired by grazing. However, this reflects the importance of diversity and composition due to the negative impacts of composition (Figure 5) and the reduced diversity by grazing (Figure 3A), which further indicates that the positive effect of increasing complexity could not offset the effect of decreasing diversity and the negative impacts of composition (Supplementary Figure 8).

Collectively, our results showed that the bacterial diversity, network complexity, and stability have enhanced the soil ecosystem’s multifunctionality, while the turnover of bacterial and fungal compositions induced by grazing can weaken the soil ecosystem’s multifunctionality (Supplementary Figure 8). It is expected to further maintain the function of the grazing grassland soil ecosystem at a higher level by optimizing the availability of soil nutrients and physical environment and increasing the niche width of bacteria and fungi, thereby improving the bacterial diversity, network complexity, and stability. Moreover, further studies with the operation of bacterial and fungal combinations will also assist to obtain a higher ecosystem function and update knowledge on the behind cause-and-effect mechanisms.

## Conclusion

Grazing generally affects subtropical grasslands. In line with our three assumptions, our evidence showed that grazing decreased soil multifunctionality. Grazing shifted the soil bacterial and fungal composition and function and reduced the soil bacterial Simpson diversity and niche width. In addition, grazing enhanced the complexity and stability of the bacterial and fungal networks. Furthermore, the bacterial Simpson diversity, network complexity, and stability had positive effects, whereas bacterial and fungal compositions had negative effects on multifunctionality. Our study provides a novel comparison of the roles of soil microbial diversity, composition, network complexity, and stability on multifunctionality, and this will improve our understanding of the multidimensions of biotic driving forces of multifunctionality. Indeed, our results may occur in improving multifunctionality through directing future efforts to optimize the composition of soil bacteria and fungi and increase the diversity and the network complexity and stability of bacteria.

## Data availability statement

The data presented in this study are deposited in the FigShare and NCBI repository, accession number: 10.6084/m9.figshare.21671246 for FigShare, PRJNA908246 for NCBI.

## Author contributions

LD: formal analysis, conceptualization, and visualization. LD, YZ, and PW: funding acquisition. LD and PW: investigation. PW: project administration. LD and LT: writing – original draft. LD, LT, JL, YZ, MW, and PW: writing – review and editing. All authors contributed to the article and approved the submitted version.

## Footnotes

1
https://github.com/dlltargeting/evcmdl

2
https://github.com/brendanf/FUNGuildR

3
https://cloud.r-project.org/bin/windows/base/old/3.6.1/R-3.6.1-win.exe

4
https://cloud.r-project.org/bin/windows/base/old/4.0.5/R-4.0.5-win.exe

5
https://gephi.org/users/download/

6
https://www.sciencedirect.com/topics/immunology-and-microbiology/intracellular-parasite
