# Supplementary material for: Grazing lowers soil multifunctionality but boosts soil microbial network complexity and stability in a subtropical grassland of China
Source: Front Microbiol. 2023 Jan 5;13:1027097. doi: 10.3389/fmicb.2022.1027097 (PMC9849757; doi:10.3389/fmicb.2022.1027097)
Supplement: Supplementary file 1 [file Data_Sheet_1.ZIP › Fig.S7.pdf]

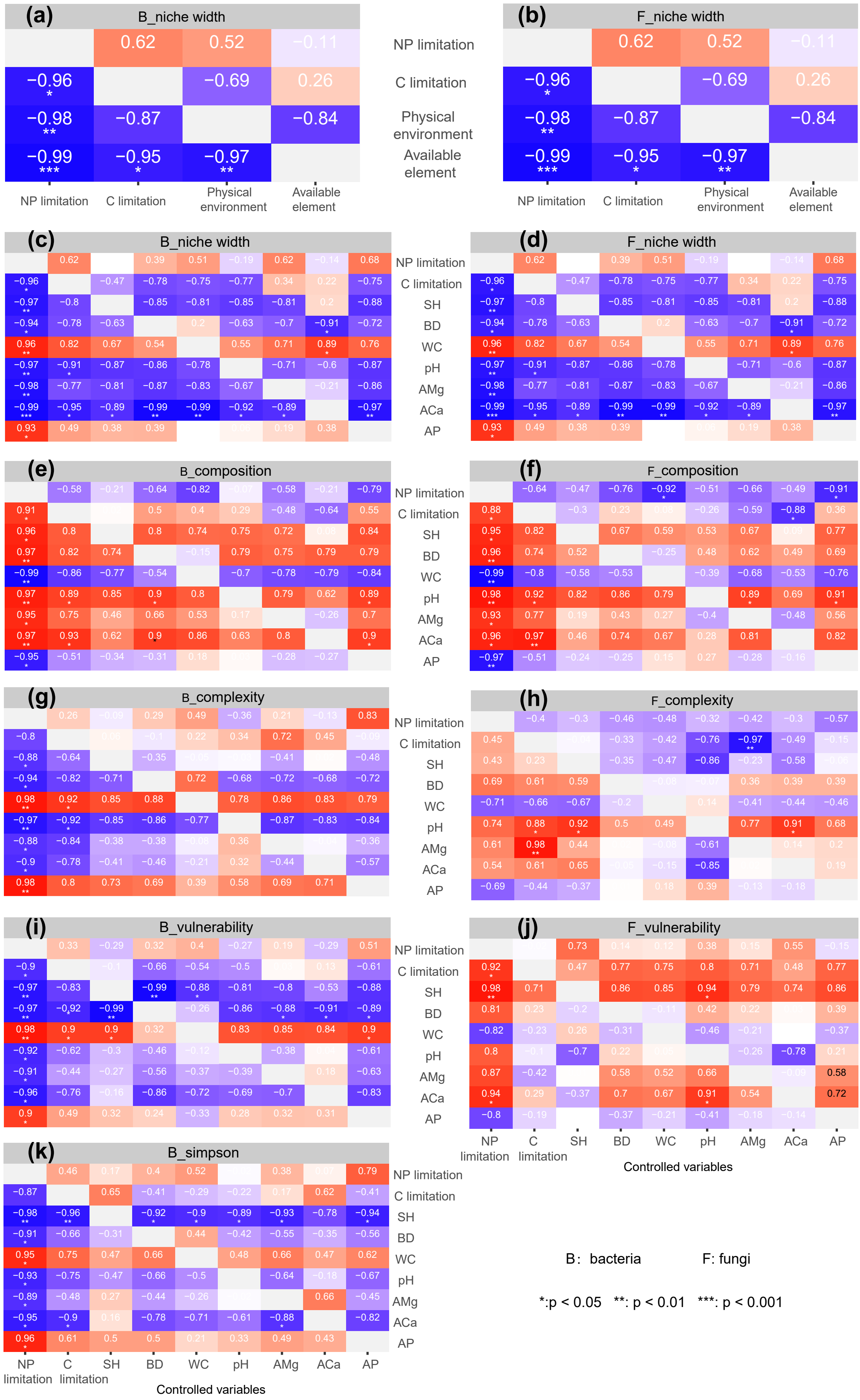
(a) B_niche width
(b) F_niche width
NP limitation
C limitation
Physical environment
Available element
(c) B_niche width
(d) F_niche width
(e) B_composition
(f) F_composition
(g) B_complexity
(h) F_complexity
(i) B_vulnerability
(j) F_vulnerability
(k) B_simpson
NP limitation
C limitation
SH
BD
WC
pH
AMg
ACa
AP
Controlled variables
B: bacteria
F: fungi
*:p < 0.05 **: p < 0.01 ***: p < 0.001
