# Supplementary material for: Grazing lowers soil multifunctionality but boosts soil microbial network complexity and stability in a subtropical grassland of China
Source: Front Microbiol. 2023 Jan 5;13:1027097. doi: 10.3389/fmicb.2022.1027097 (PMC9849757; doi:10.3389/fmicb.2022.1027097)
Supplement: Supplementary file 1 [file Data_Sheet_1.ZIP › Fig.S8.pdf]

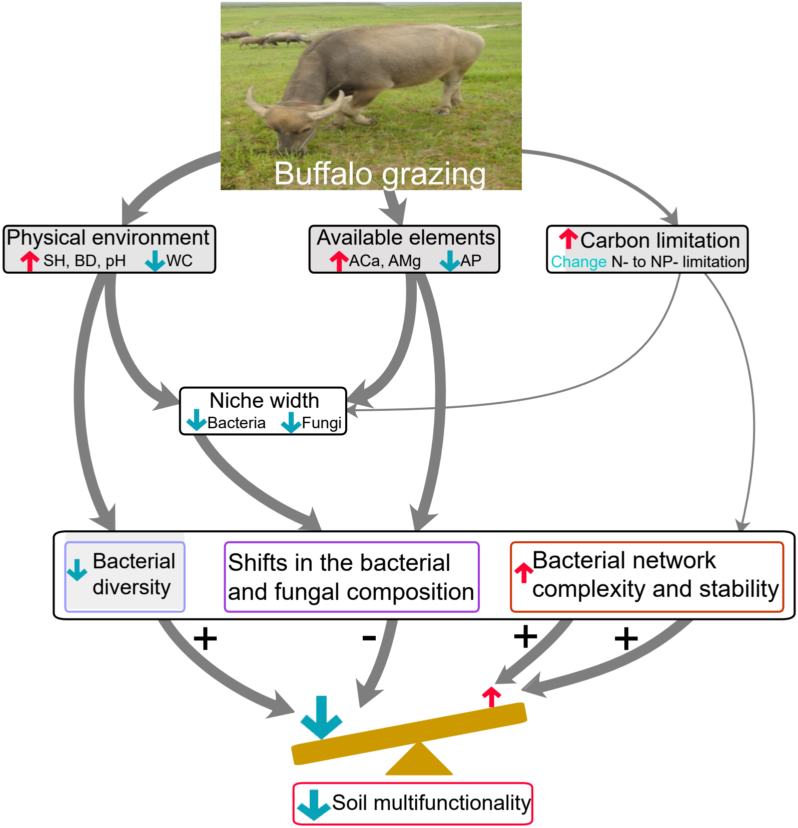
Buffalo grazing
Physical environment
SH, BD, pH
WC
Available elements
ACa, AMg
AP
Carbon limitation
Change N- to NP- limitation
Niche width
Bacteria
Fungi
Bacterial diversity
Shifts in the bacterial and fungal composition
Bacterial network complexity and stability
+
-
+
+
Soil multifunctionality
