# Supplementary material for: Grazing lowers soil multifunctionality but boosts soil microbial network complexity and stability in a subtropical grassland of China
Source: Front Microbiol. 2023 Jan 5;13:1027097. doi: 10.3389/fmicb.2022.1027097 (PMC9849757; doi:10.3389/fmicb.2022.1027097)
Supplement: Supplementary file 1 [file Data_Sheet_1.ZIP › Legends for Fig.S1-S8.docx]

**Fig.S1** Soil chemical property, stoichiometric ratio, enzyme activity, and nitrogen/phosphorus limitation under grazed and ungrazed

**Fig.S2** Soil bacterial (a, c, e, g) and fungal (b, d, f, h, i) diversity under grazed and ungrazed

**Fig.S3** Principal component analysis (PCA, a, b) and Nonmetric multidimensional scaling (NMDS, c, d) showing a clear distinction for soil microbial composition (OTUs level) between grazed and ungrazed

**Fig.S4** Soil bacterial (a-e) and fungal (f-g) putative functions under grazed and ungrazed

**Fig.S5** Differences in niche breadth (a), and assembly mechanism (b, c) of bacterial (b) and fungal (c) communities between grazed and ungrazed

**Fig.S6** Zero order (a-b) and first order (c-i) Pearson correlation analysis showing the relationships of driving forces and soil microbial niche width, composition, diversity, and network.

**Fig.S7** First order (a-k) Pearson correlation analysis showing the relationships of driving forces and soil microbial niche width, composition, diversity, and network. SH, soil hardness; BD, bulk density; WC, water content; ACa, calcium availability; AMg, magnesium availability; AP, Phosphorus availability

**Fig.S8** The conceptual model diagram showing that grazing changed soil available element, physical environment, microbial C limitation, and microbial niche width, consequent the bacterial and fungal compositions and the bacterial diversity, network complexity and stability. The bacterial diversity, network complexity and stability have enhanced the soil ecosystem multifunctionality, while the turnover of bacterial and fungal compositions can weaken the soil ecosystem multifunctionality. However, the positive effect of increasing complexity could not offset the effect of decreasing diversity and the negative impacts of composition, possibly resulted in the decline of the soil ecosystem multifunctionality. SH, soil hardness; BD, bulk density; WC, water content; ACa, available calcium content; AMg, available magnesium content; AP, available phosphorus. N, nitrogen; P, phosphorus.
