# Supplementary figures and images for: Grazing lowers soil multifunctionality but boosts soil microbial network complexity and stability in a subtropical grassland of China
Source: Front Microbiol. 2023 Jan 5;13:1027097. doi: 10.3389/fmicb.2022.1027097 (PMC9849757; doi:10.3389/fmicb.2022.1027097)

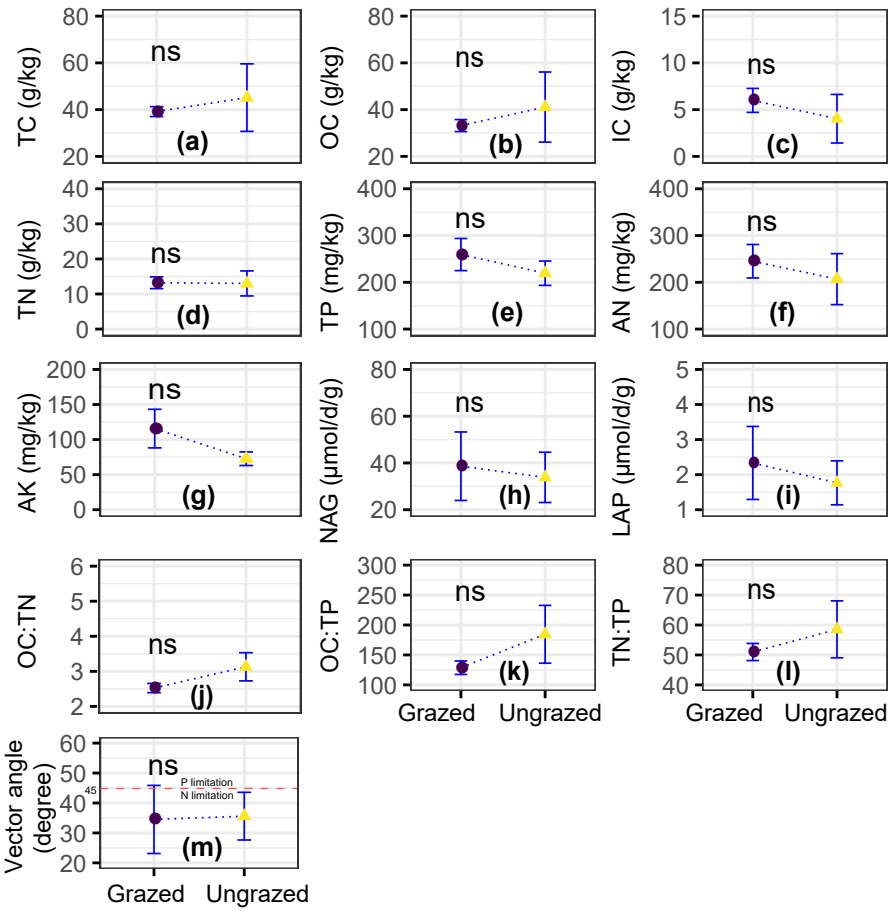

Supplement: Supplementary file 1 [file Data_Sheet_1.ZIP › Fig.S1.pdf]

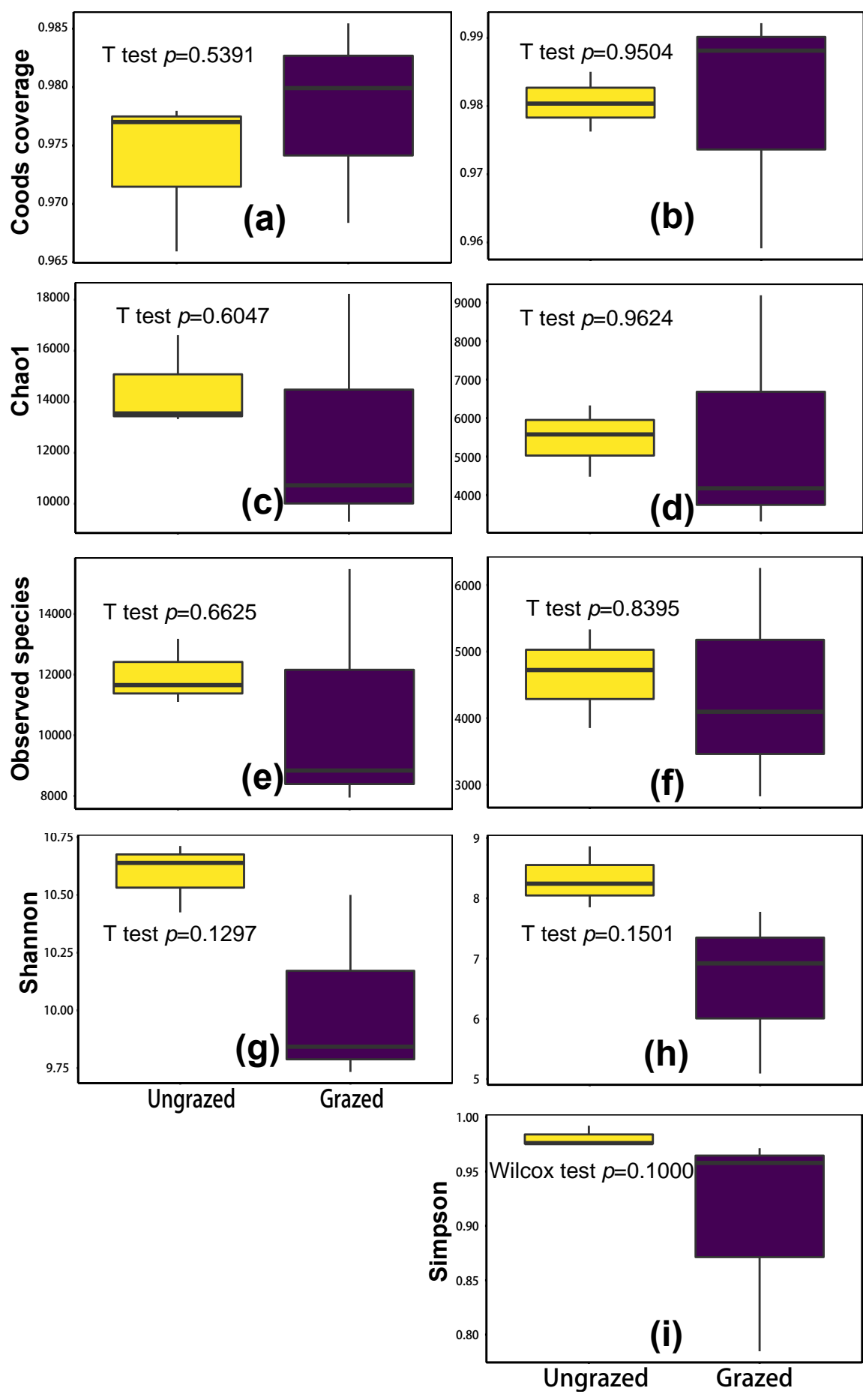

Supplement: Supplementary file 1 [file Data_Sheet_1.ZIP › Fig.S2.pdf]

# Bacteria

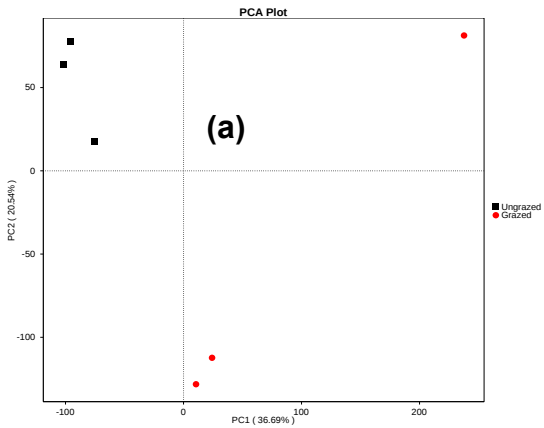

# Fungi

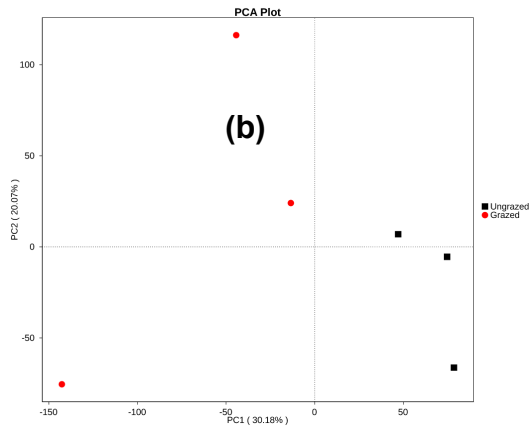

NMDS - MDS1 vs MDS2

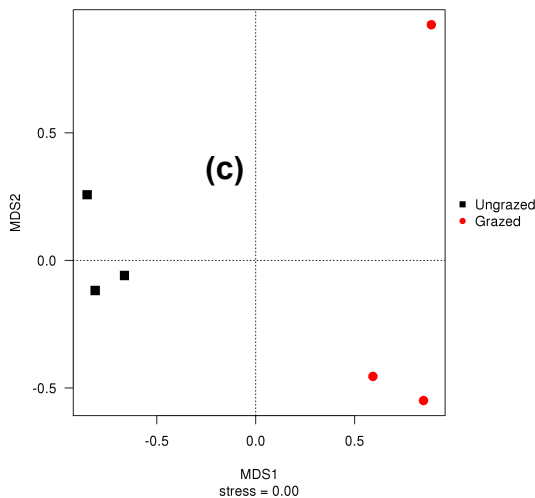

NMDS - MDS1 vs MDS2

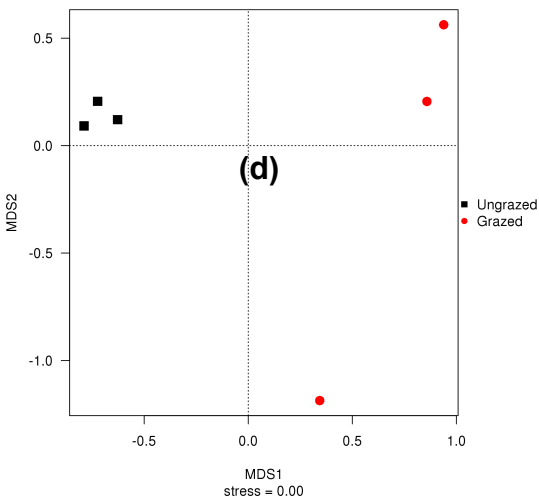

Supplement: Supplementary file 1 [file Data_Sheet_1.ZIP › Fig.S3.pdf]

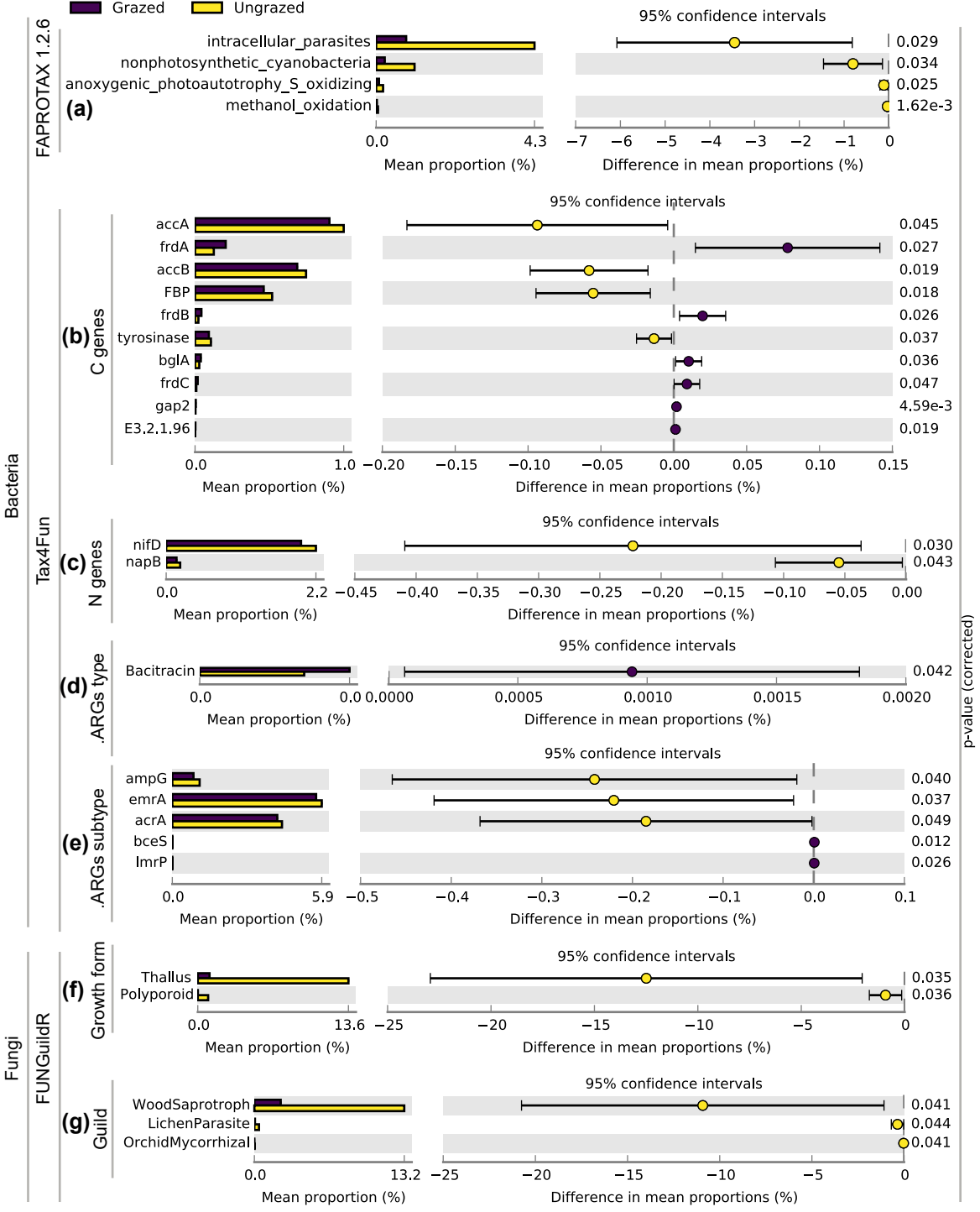

Supplement: Supplementary file 1 [file Data_Sheet_1.ZIP › Fig.S4.pdf]

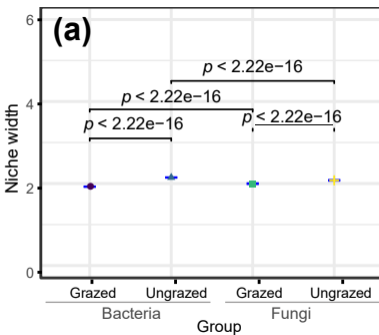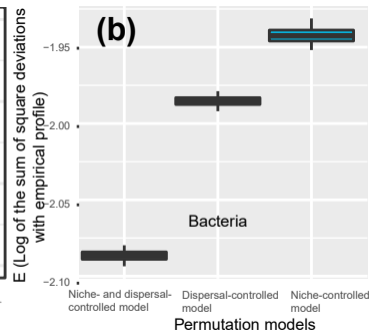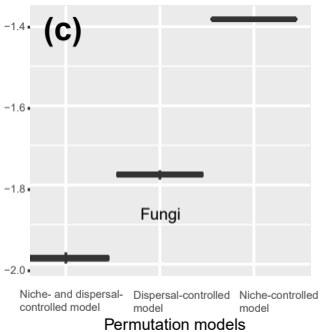

Supplement: Supplementary file 1 [file Data_Sheet_1.ZIP › Fig.S5.pdf]

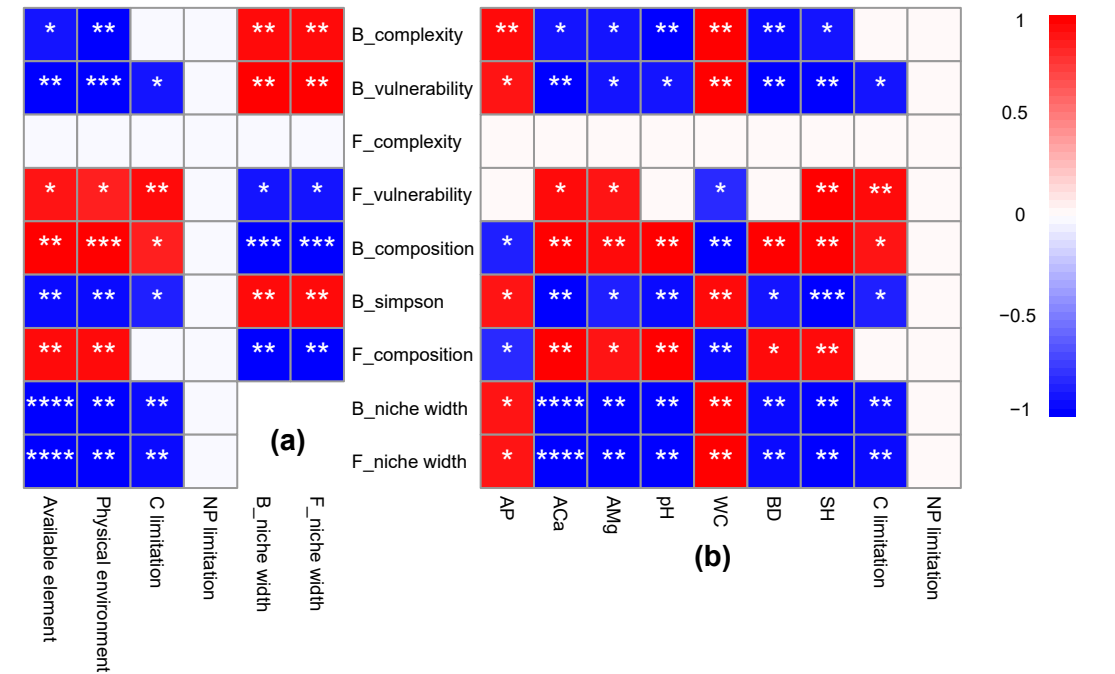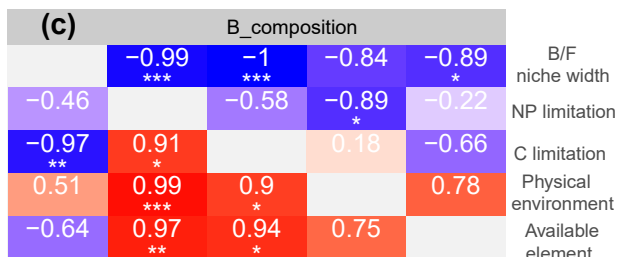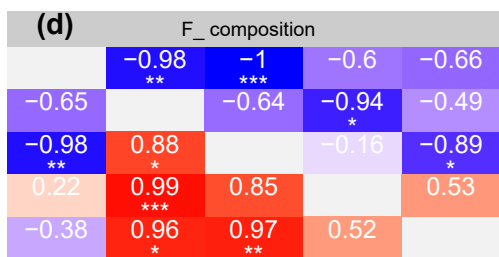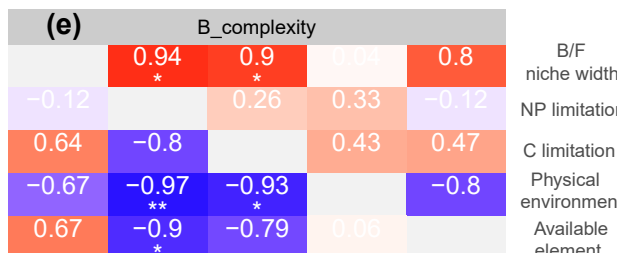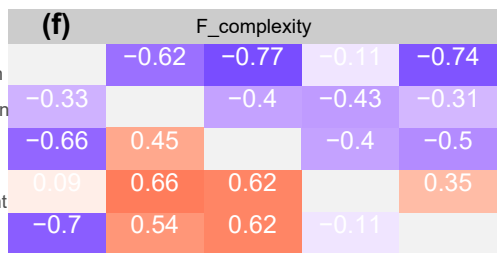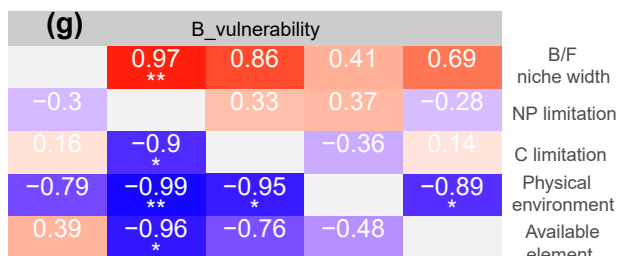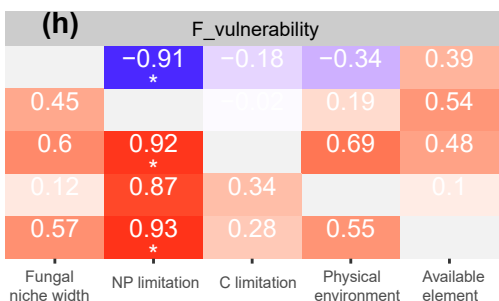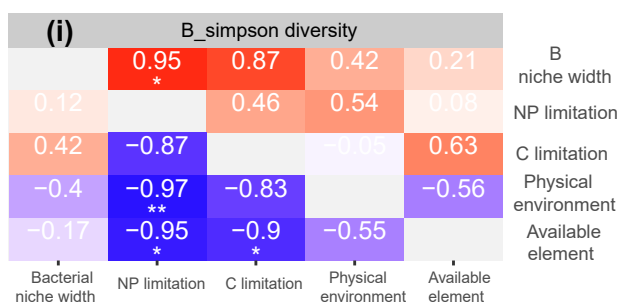

Controlled variables

B: bacteria

F: fungi

\*: p < 0.05 \*\* : p < 0.01 \*\*\*: p < 0.001

Supplement: Supplementary file 1 [file Data_Sheet_1.ZIP › Fig.S6.pdf]
